# 

**Published:** 1866-10

**Authors:** 


### Books and Pamphlets Received.

Elements of Medical Chemistry. By B. Howard Rand, M. D., Professor of Chemistry in Jefferson Medical College. Philadelphia; T. Elwood Zell & Co., 1867.

Therapeutics of Zymotic Diseases. By Edward B. Stevens, M. D., of Cincinnati.

A Practical Treatise on Fractures and Dislocations. By FRANK HASTINGS HAMILTON, A. B., A. M., M. D., Professor of the Principles of Surgery, Military Surgery and Hygiene, and of Fractures and Dislocations in Bellevue Hospital Medical College; Surgeon to Bellevue Hospital and to the Charity Hospital, New York; Professor of Military Surgery, etc., in the Long Island College Hospital; author of a Treatise on Military Surgery. Third edition, revised and improved. Illustrated with 294 wood cuts. Philadelphia: Henry C. Lea.

A Practical Treatise on the Physical Exploration of the Chest, and the Diagnosis of Diseases affecting the Respiratory Organs. By AUSTIN FLINT, M. D., Professor of the Principles and Practice of Medicine in the Bellevue Hospital Medical College, and in the Long Island College Hospital; Fellow of the New York Academy of Medicine, etc. Second edition, revised. Philadelphia: Henry C. Lea.

A Hand-Book of Ophthalmic Surgery for the use of Practitioners. By JOHN Z. LAURENCE, F. R. C. S., M. A. (Unv. Lond.,) Surgeon to the Ophthalmic Hospital, Southwark; Editor of the Ophthalmic Review; Member of the Heidelberg Ophthalmological Society, of the Universal Ophthalmological Society, of the Pathological Society of London, of the Harveian Society, of the Society of Practical Medicine of Paris, of the Society of German Naturalists and Physicians, and ROBERT C. MOON, House Surgeon to the Ophthalmic Hospital, Southwark. With numerous illustrations. Philadelphia: Henry C. Lea.

A Practical Treatise on Diseases of the Skin. By J. MOORE NELIGAN, M. D., M. R. I. A., etc. Fifth American from the second revised and enlarged Dublin edition. By T. W. BELCHER, M. A., M. D., Dublin; Fellow, Censor. Examiner in Materia Medica and Medical Jurisprudence, and in Arts, and Honorary Librarian, King and Queen's College of Physicians in Ireland; Honorary Member of the Cork Medical Society; Physician to the Dublin Dispensary for Skin Diseases; and sometime one of the Physicians to the Cork Fever Hospital. Philadelphia: Henry C. Lea.

Transactions of the Twenty-First Annual Annual Meeting of the Ohio Medical Society, held at Ohio White Sulphur Springs, July 19th, 20th and 21st, 1866.

### Exchange Journals.

The following Journals are regularly received in exchange:

London Lancet—Editors, J. H. Bennett, M. D., T. Wakely, jr., M. R. C. S. E.

Braithwaite's Retrospect of Practical Medicine and Surgery. New York: W. A. Townsend, 434 Broome street.

The Ophthalmic Review, edited by J. Z. Lawrence and Thomas Winslow, London.

American Journal of the Medical Sciences, edited by Isaac Hays, M. D.

Boston Medical and Surgical Journal, edited by Samuel L. Abbott, M. D. and James C. White, M. D.

The American Journal of Insanity, edited by the officers of the New York State Lunatic Asylum.

The New York Medical Journal.

The Cincinnati Lancet and Observer, edited by Edward B. Stevens, M. D. and John A. Murphy, M. D.

The St. Louis Medical and Surgical Journal, edited by M. L. Linton, M. D. and Frank W. White, M. D.

The Medical Record.

The Chicago Medical Journal.

The Chicago Medical Examiner, edited by N. S. Davis, M. D..

The Medical and Surgical Reporter, edited by S. W. Butler, M. D.

The Cincinnati Journal of Medicine, edited by George C. Blackman, M. D., Theophilus Parvin, M. D. and Roberts Bartholow, M. D.

The Richmond Medical Journal, edited by E. S. Gaillard, M. D. and W. S. McChesney, M. D.

The Savannah Journal of Medicine, edited by Juria Harris, M. D., J. B. Read· M. D. and J. G. Thomas, M. D.

The Medical Reporter, edited by J. S. B. Alleyne, M. D. and O. F. Potter, M. D.

The Detreit Review of Medicine and Pharmacy, edited by George T. Andrews, M. D., S. T. Duffield, M. D. and E. W. Jenks, M. D.

Atlanta Medical and Surgical Journal, edited by J. G. Westmoreland, M. D.

The Medical and Surgical Monthly, Memphis, Tenn., edited by Frank A. Ramsay, M. D., D. D. Saunders, M. D., E. Mills Willett, M. D. and William H. White, M. D.

Southern Journal of Medical Sciences, edited by E. D. Turner, M. D., D. Warren Brickell, M. D. and C. Beard, M. D.

The Pacific Medical and Surgical Journal and Press, edited by Henry Gibbons, M. D.

The Galveston Medical Journal, edited by Greenvill Dowell, M. D.

The New Orleans Medical Record, a semi-monthly Journal of the Medical Sciences, edited by Bennett Dowler, M. D. and S. R. Chambers, M, D.

The American Journal of Pharmacy, edited by William Proctor, jr.

Medical News and Library, by Henry C. Lea, Philadelphia.

The Druggist's Circular and Chemical Gazette; The Journal of Materia Medica, by Joseph Bates, M. D. and H. A. Tilden.

The Dental Cosmos, edited by J. H. McQuillen, D. D. S. and George J. Zeigler, M. D.

The Atlantic Monthly, published by Ticknor, Fields & Co., Boston.

Godey's Lady's Book, published by Louis A. Godey, Philadelphia.

The Herald of Health.

Eclectic Medical and Surgical Journal, Philadelphia.

Eclectic Medical Journal, Cincinnati.

American Eclectic Medical Review, New York.

Eclectic Medical Journal, Cincinnati, Ohio.

Philadelphia University Journal.

Dental Register, Cincinnati.

The Nation.

Every Saturday, published by Ticknor, Fields & Co., Boston.

Educational Monthly.

